# *Pseudomonas aeruginosa* assembles H1-T6SS in response to physical and chemical damage of the outer membrane

**DOI:** 10.1126/sciadv.adr1713

**Published:** 2025-03-05

**Authors:** Mitchell Brüderlin, Maxim Kolesnikov, Florian Röthlin, Roderick Y. H. Lim, Marek Basler

**Affiliations:** ^1^ Biozentrum, University of Basel, Spitalstrasse 41, 4056 Basel, Switzerland.; ^2^ Swiss Nanoscience Institute, University of Basel, Klingelbergstrasse 82, 4056 Basel, Switzerland.

## Abstract

Bacteria respond to environmental stimuli and attacks from competing organisms. *Pseudomonas aeruginosa* assembles the type VI secretion system (H1-T6SS) to precisely retaliate against aggressive competing bacteria. However, we lack an understanding of how the H1-T6SS assembly dynamically responds to nanomechanical forces. To address this, we analyzed live cells using correlative atomic force microscopy (AFM) and fluorescence microscopy. We show that indentation forces above 7 nanonewtons trigger local, repeated and targeted H1-T6SS assemblies within seconds of impact by the AFM tip. Analysis of the corresponding AFM force curves shows that a breach of a single layer of the cell envelope is necessary and sufficient for triggering H1-T6SS assembly. Accordingly, polymyxin B nonapeptide, which damages the outer membrane, also triggers H1-T6SS assembly. This suggests that *P. aeruginosa* has evolved a danger-sensing mechanism that enables rapid and precise deployment of its antibacterial H1-T6SS in response to breaches in the outer membrane.

## Teaser

The assembly of the *P. aeruginosa* H1-T6SS is triggered by outer-membrane damage induced chemically or mechanically by an AFM tip.

## INTRODUCTION

Bacteria use sensory mechanisms that regulate signal transduction and gene expression, enabling them to adapt to environmental stimuli within minutes (*1*). These sensors also trigger posttranslational modifications that regulate protein activity, localization, and the assembly of protein complexes (*2*). Such regulatory systems are crucial for bacterial survival, enabling them to compete for resources or infect higher organisms, causing disease. They include biosynthetic pathways that produce antibiotics inhibiting the growth of competing organisms (*3*) or secretion systems that deliver toxins into neighboring cells (*4*). However, the mechanisms by which bacteria establish thresholds for external stimuli and calibrate their responses to optimize resource use remain poorly understood.

A notable example of protein complex regulation is the assembly of the type 6 secretion system (T6SS), which Gram-negative bacteria use to attack and deliver toxins into bacterial and eukaryotic cells (*5*, *6*). The T6SS consists of an envelope-spanning protein complex (TssM/TssL/TssJ) that acts as an anchor (*7*), a connected cytoplasmic baseplate (TssE/TssG/TssF/TssK) (*8*), and a long contractile sheath (TssB/TssC) assembled around an inner tube (Hcp) (*9*–*12*). The tube is capped by a needle-like spike (VgrG/PAAR) assembled in the center of the baseplate (*13*–*15*). The sheath-tube polymer is often assembled across the whole bacterial cytoplasm. When the sheath contracts, it pushes the inner tube with the spike and associated effectors through the baseplate and the membrane complex out of the bacterium and into the environment or a neighboring cell (*16*). The subunits of the contracted sheath are then unfolded by the adenosine triphosphatase (ATPase) ClpV to allow new cycles of T6SS assembly (*17*, *18*).

In some bacteria, such as *Vibrio cholerae*, T6SS assembly occurs randomly without any obvious cue, suggesting that it is regulated solely on a transcriptional level (*19*). In other bacteria, T6SS gene clusters encode accessory proteins that are important for regulating the rapid assembly of the T6SS in response to a specific signal (*20*). For example, phosphorylation of Fha in *Pseudomonas aeruginosa* (*21*) or TssL in *Agrobacterium tumefaciens* was shown to increase T6SS assembly rate (*22*). Removing TagF in *P. aeruginosa* or *Serratia marcescens* leads to hyperactivation of the T6SS, suggesting that TagF plays a role in inhibiting T6SS assembly (*23*, *24*). *Acinetobacter* and *Burkholderia thailandensis* position their T6SS assemblies to a site of cell-cell contact using TslA and OmpA, enabling a targeted response and increasing the efficiency of toxin translocation into neighboring cells (*25*).

**Fig. 1.**
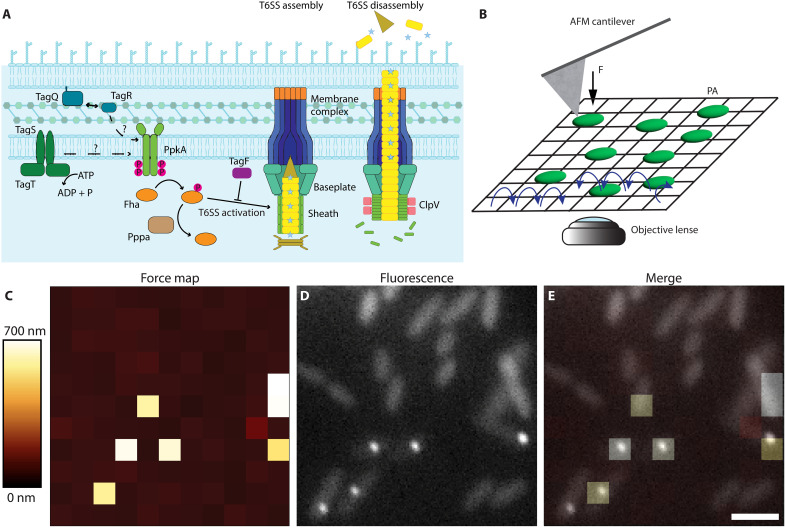
Mechanical force triggers H1-T6SS assembly in *P. aeruginosa* cells. (**A**) Simplified model of H1-T6SS regulation and assembly. The proteins known to be required for initiation of T6SS assembly include TagQ, TagR, TagS, TagT, Fha, and PpkA. Inhibition and deactivation are mediated by TagF and PppA, respectively. The unfoldase (ClpV) is responsible for disassembly of the contracted sheath. (**B**) The green rods represent *P. aeruginosa* (PA) cells, and the blue arrows show the path of the AFM tip during a force mapping experiment. The direction of applied force exerted by the AFM tip is indicated by the black arrow. (**C**) The AFM force map shows the offset height of *P. aeruginosa ∆retS*, *∆fliC*, *tssB1-mNeonGreen* cells during an indentation experiment. Bright pixels indicate force curves where the AFM detects a higher offset height value, meaning a lower approach distance before reaching the predetermined set-point force. The range of height differences over the whole map is shown in the lookup table. (**D**) The fluorescence image obtained within the same region of interest shows multiple active TssB1-mNeonGreen assemblies as indicated by the high intensity fluorescence foci in some of the cells. (**E**) The overlay of the force map with the corresponding fluorescence image shows the correlation between AFM tip indentations and T6SS assemblies. Scale bar, 2 μm. F, applied force.

Perhaps most striking is the antibacterial H1-T6SS of *P. aeruginosa*, which assembles in direct response to attacks from neighboring cells (*26*, *27*). This “Tit-for-Tat” response requires a set of accessory proteins encoded in the H1-T6SS cluster: TagQ, an outer membrane (OM) lipoprotein, the periplasmic TagR, and the inner membrane (IM) ATPase TagT and TagS (*26*–*29*). Dimerization of the inner membrane kinase PpkA results in phosphorylation of Fha (*21*), which is followed by repeated H1-T6SS assembly that is aimed with remarkable precision toward the attacking cell (Fig. 1A) (*27*). This targeted retaliation likely enables *P. aeruginosa* to respond precisely to local attacks from competing bacteria, minimizing the risk of misfiring to reduce the energy demands of assembly. H1-T6SS assembly is blocked upon Fha dephosphorylation by the phosphatase PppA (*21*). However, H1-T6SS assembly can also be triggered by membrane-damaging antibiotics or membrane-destabilizing agents (*26*, *30*, *31*). Also, inhibiting membrane biogenesis can trigger H1-T6SS assemblies because of membrane stress (*32*). Therefore, the mechanisms that *P. aeruginosa* use to activate local H1-T6SS assembly remain unresolved.

Although bulk studies on H1-T6SS assembly initiation have provided valuable insights into its dynamic regulation, they fall short in triggering H1-T6SS assembly at the single-event level in a highly localized, controllable manner. In this study, we used correlative atomic force microscopy (AFM) and fluorescence microscopy to investigate H1-T6SS assembly at the single-event level by provoking *P. aeruginosa* cells with mechanical force. AFM is a well-established technique that uses a ~5-nm sharp tip to probe the mechanical properties of bacterial cells (*33*–*36*), including *P. aeruginosa* (*37*). Here, we demonstrate that indentation forces exceeding a threshold of ~7 nN trigger rapid, repeated, and targeted H1-T6SS assemblies within seconds of AFM tip impact. This response correlates with a breach of a single envelope layer and can be similarly triggered by chemical disruption of the OM. Together, our data indicate that physical and chemical damage to the OM is both necessary and sufficient for initiating H1-T6SS assembly.

## RESULTS

### Mechanical indentation of cells triggers H1-T6SS assembly

We generated a *P. aeruginosa ∆retS*, *∆fliC*, *tssB1-mNeonGreen* strain, tagging the TssB1 sheath protein with mNeonGreen to track H1-T6SS assembly. RetS was deleted to enhance H1-T6SS cluster expression in vitro (*38*), while FliC deletion prevented flagella assembly to promote bacterial attachment to glass (*39*). We verified that this strain could efficiently outcompete the *V. cholerae T6SS^+^* strain, being dependent on *V. cholerae*’s T6SS activity, similar to the parental *P. aeruginosa ∆retS* strain (fig. S1). Thereafter, we performed AFM tip–based cell indentation in “force mapping” mode (*40*, *41*) while simultaneously tracking H1-T6SS assembly via fluorescence microscopy (Fig. 1B). This allowed us to detect H1-T6SS assembly in response to AFM indentation (Fig. 1, C to E, and movie S1). Specifically, AFM force mapping involves acquiring an array of individual force curves within a defined area of interest where the cells reside, typically spanning 15 μm by 15 μm to 30 μm by 30 μm (Fig. 1B). Each array of force curves then generates a quasi-topographical map, or “force map,” which displays the relative distance (height) traveled by the AFM tip to reach its predetermined force set point (Fig. 1C). To benchmark our method, AFM force mapping yielded an elasticity of 788.7 ± 431.6 kPa for individual cells using the modified Hertz-Sneddon model (fig. S2) (*42*), consistent with previous AFM studies on *P. aeruginosa* (*37*, *43*, *44*).

We next assessed the size and shape of the TssB1-mNeonGreen foci to ascertain whether the H1-T6SS assembly was influenced by the approach direction of the AFM tip, which was perpendicular to the imaging plane (fig. S3). We reasoned that assemblies oriented toward the AFM tip (pointing out-of-plane) would appear as round, diffraction limited foci, while assemblies aligned tangentially to the tip approach (in-plane) would produce elongated fluorescent structures. To differentiate between these orientations, we fit ellipses to the fluorescent foci and calculated the ratio of their minor to major axes (fig. S3). As a conservative estimate, foci with ratios above 1.25 were considered as in-plane, while those below were considered out-of-plane. The analysis showed that 78.6% of all assemblies were oriented out-of-plane, suggesting that H1-T6SS assembly is mechanically triggered and aligns preferentially with the AFM tip’s approach direction.

To confirm that AFM indentation activates H1-T6SS through the same signaling pathway observed in competition assays, we deleted the TagQ and PpkA genes in the parental strain, both of which are essential for the retaliatory phenotype (*27*, *31*). Notably, no H1-T6SS assemblies were detected upon AFM indentation in these mutants, although occasional assemblies independent of AFM indentation were observed, confirming that the cells were still capable of H1-T6SS assembly (movie S2). As expected, *P. aeruginosa ∆retS ∆fliC*, *tssB1-mNeonGreen*, *∆tagQ* and *P. aeruginosa ∆retS ∆fliC*, *tssB1-mNeonGreen*, *∆ppkA* strains failed to outcompete T6SS-positive *V. cholerae* similar to a negative control strain *P. aeruginosa ∆retS ∆fliC*, *tssB1-mNeonGreen*, *∆tssM* (fig. S1).

**Fig. 2.**
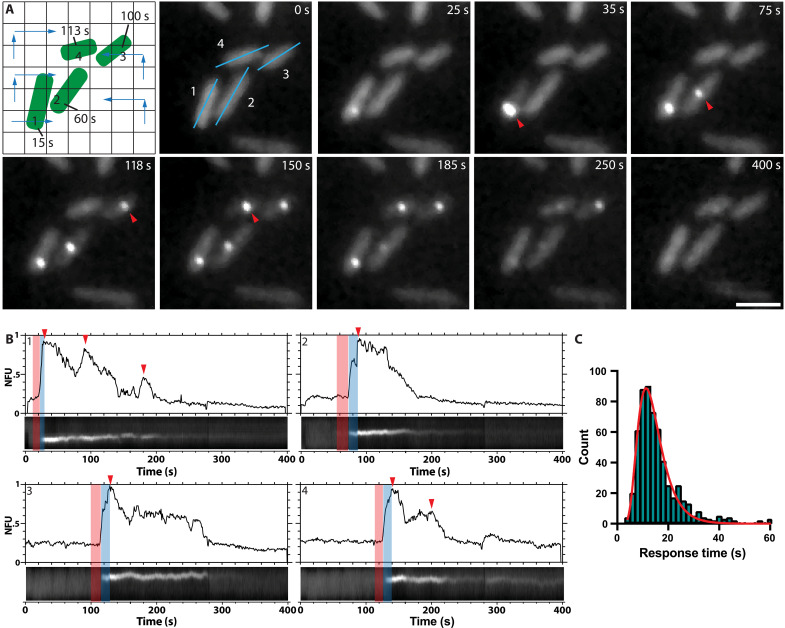
Characteristic timescales of *P. aeruginosa* H1-T6SS activation and assembly. (**A**) The path taken by the AFM tip (blue arrows) is shown in the first panel. Each cell is indented at a specific position (numbered grid points) and time (time stamp). Images taken from a time lapse (400 s) show the cells with fluorescent foci (TssB1-mNeonGreen) in *P. aeruginosa ∆retS*, *∆fliC*, *tssB1-mNeonGreen* cells that assembled upon being indented by the AFM tip. The red arrowheads indicate the four assemblies shown in the cartoon. The fluorescent foci disassembled by the end of the time-lapse. Cells 1 and 2 display an assembly parallel to the sample plane. Cells 3 and 4 show assembly perpendicular to the image plane (see fig. S3 for more details). The blue lines in the first image frame indicate the kymographs in (B). Scale bar, 1 μm. (**B**) The kymographs display H1-T6SS assemblies in *P. aeruginosa ∆retS*, *∆fliC*, *tssB1-mNeonGreen* cells imaged over the course of 400 s. The intensity profiles above the kymographs, reported in normalized fluorescence units (NFU), illustrate increases in fluorescence, indicating repeated H1-T6SS assemblies (red arrowheads). The response time is defined as the interval between the moment of AFM tip contact and a marked increase in fluorescence (red bar). The assembly time is defined as the interval between start of fluorescence increase and visible contraction (blue bar). (**C**) A log-normal fit (red line) to the distribution of H1-T6SS assembly response times (*N* = 68, *n* = 584) yields a mean response time of 13.2 ± 1.5 s.

As the AFM tip appears as a dark spot in the fluorescence image (fig. S4), its motion can be accurately tracked, enabling synchronization between the moment of AFM tip contact with a cell and H1-T6SS assembly in the corresponding fluorescence data (Fig. 2A and movie S3). Subsequently, we analyzed 125 time-lapse series and generated kymographs to visualize variations in TssB1-mNeonGreen fluorescence intensity (Fig. 2B). Each kymograph corresponds to a single H1-T6SS focus within an individual cell. The time between AFM indentation and initial fluorescence increase denoting H1-T6SS assembly is defined as the “response time.” A single AFM indentation triggered an initial assembly with mean response time of 13.2 ± 1.5 s, followed by one to seven consecutive H1-T6SS assemblies, resulting in an overall duration of 151 ± 99 s before assembly ceased, restoring baseline fluorescence (Fig. 2, B and C). This is consistent with previous observations that *P. aeruginosa* responds to T6SS attacks by repeatedly assembling the H1-T6SS (*45*). Additionally, we ascertained a mean H1-T6SS sheath assembly time of 9 ± 3 s, defined as the time interval between initial fluorescence detection and sheath contraction (Fig. 2B). These findings demonstrate that repeated *P. aeruginosa* H1-T6SS assemblies can be initiated by external forces in just over 10 s and that the H1-T6SS response relies on the TagQRST/PpkA signaling cascade.

### H1-T6SS assembly is triggered by a threshold loading force

**Fig. 3.**
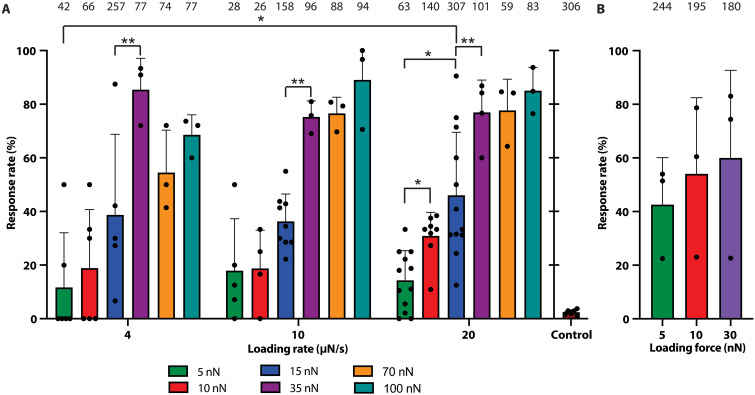
Response rate of H1-T6SS assembly as a function of loading rate and force. (**A**) Comparison of T6SS assembly response rates across three loading rates. The bar plots show the ratio of cells that form T6SS assemblies to the number of indented cells, aggregated over all tested conditions. For each condition, biological replicates ≥ 3 and number of hits > 25. The numbers above each bar denote the number of successful AFM indentations. The statistical significance between pairs of conditions was assessed using one-way analysis of variance (ANOVA), with asterisks marking the most notable differences (*P < 0.05 and **P < 0.01). The baseline H1-T6SS activity in a 30 μm–by–30 μm area over the course of 80 s is plotted separately as a control. (**B**) T6SS assembly response rates obtained from a dense monolayer of bacteria measured at a loading rate of 20 μN/s across three loading forces.

To better understand the sensing mechanism of *P. aeruginosa*, we studied H1-T6SS assembly over different loading forces (5, 10, 15, 35, 70, and 100 nN) and force loading rates (4, 10, and 20 μN/s). The latter is defined as the speed at which force is applied to a cell (*46*). To allow for comparisons across different conditions, we calculated the response rate, defined as the ratio of H1-T6SS assemblies observed via fluorescence microscopy to the number of cells indented by the AFM tip in each force map. In the absence of AFM indentation, the baseline H1-T6SS activity of our strain was 2.35 ± 0.94 %. Between 5 and 15 nN, the response rate increased with force and was significantly higher than the baseline activity, reaching a maximum of ~40% across the three loading rates (Fig. 3A). We subsequently found that applying higher forces markedly increased the response rate to 85%, without significant variations between 35, 70, and 100 nN across the three loading rates (see movie S4). This analysis indicates that the mechanism for triggering H1-T6SS assembly is more sensitive to the loading force than to the loading rate.

Subsequently, seeding cells at a higher density led to increased response rates at lower forces (Fig. 3B and movie S5), likely due to the higher cell density restricting cell movement, increasing the chances of successful indentation. Overall, our data indicate that H1-T6SS initiation and assembly in *P. aeruginosa* is responsive to loading force.

### OM rupture triggers H1-T6SS assembly

**Fig. 4.**
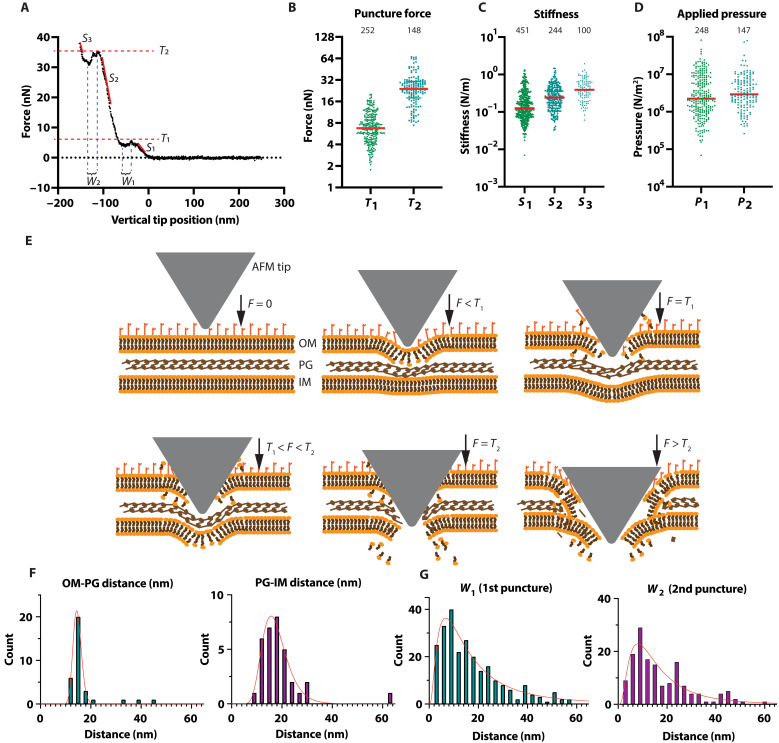
Force curve analysis and model of indentation for high force regime. (**A**) A force curve showing two puncture thresholds, *T*_1_ and *T*_2_ (red dashes), and different stiffnesses denoted as *S*_1_, *S*_2_, and *S*_3_ with corresponding slopes (red lines). *S_1_* and *S_2_* represent sample stiffnesses before the first and second puncture, respectively. *S_3_* represents the sample stiffness following the second puncture. Thicknesses of the broken barriers, *W*_1_ and *W*_2_, are also indicated (see fig. S6 for additional data). The displayed curve was obtained at an applied force and loading rate of 35 nN and 20 µN/s, respectively. (**B**) Median puncture forces are given by *T*_1_ = 6.8 nN for the first barrier and *T*_2_ = 23.9 nN for the second (red lines). (**C**) Median stiffness values are given by *S_1_* = 0.13 N/m, *S_2_* = 0.24 N/m and *S_3_* = 0.39 N/m (red lines). (**D**) The pressure applied by the AFM tip has median values of 2.24 × 10^6^ N/m^2^ and 2.91 × 10^6^ N/m^2^ for the first and second punctures, respectively (red lines). (**E**) The model illustrates the AFM tip trajectory through the cell envelope, with downward arrows representing the force applied during indentation. See text for details. (**F**) The distances calculated from cryo–electron microscopy images of the *P. aeruginosa ∆retS*, *∆fliC*, *tssB1-mNeonGreen* cell envelope (fig. S7; *n* = 37). The average spacings are 15.9 ± 5.6 nm for the OM-PG and 18.0 ± 5.1 nm for the PG-IM, respectively. (**G**) The distances of *W*_1_ and *W*_2_ correspond to 17.0 ± 12.6 nm (*n* = 248) and 17.4 ± 12.0 nm (*n* = 150), respectively. F, force.

Next, we analyzed the force curves to better understand how increasing the loading force correlates with the triggering of H1-T6SS assembly (Fig. 4). We noticed that 28% of all force curves returned to the zero-force baseline upon initial indentation before making hardwall contact with the underlying glass substrate (fig. S5). Upon closer inspection, these measurements corresponded to cells that had been displaced by the AFM tip and were omitted from further analysis. The remaining 72% of force curves displayed regions of increasing stiffness (*S*_1_ < *S*_2_ < *S*_3_), followed by abrupt reductions in force, suggesting potential membrane puncture or breakage events, consistent with previous studies of *E. coli* (Fig. 4, A to C) (*47*). We then hypothesized that the first puncture event (*T*_1_), occurring at a median threshold of 6.8 nN (observed in 60% of examined force curves), represents the puncture of the cell OM. This was followed by a second puncture event (*T*_2_), at a median value of 23.9 nN (in 48% of examined curves), likely resulting from the compression (*S*_2_) and subsequent rupture of the peptidoglycan layer (PG) and the inner cell membrane (IM) (Fig. 4E and fig. S6). Given *T*_1_ and *T*_2_, along with their respective indentation depths, we estimated the median pressures required for the two puncture events (Eq. 1; see Materials and Methods), yielding 2.24 × 10^6^ N/m^2^ for *T*_1_ and 2.91 × 10^6^ N/m^2^ for *T*_2_ (Fig. 4D). Note that these values are comparable to the estimated pressure of 5.7 × 10^6^ N/m^2^ exerted by the injection needle of R-type pyocin to puncture the cell envelope of *P. aeruginosa* (*48*). Hence, the increase in the response rate for H1-T6SS assembly at forces exceeding *T*_1_ and *T*_2_ (Fig. 4A) suggests that puncturing one or both membranes is necessary to initiate H1-T6SS assembly.

To validate this model, we used cryo–electron microscopy to measure the spacing between layers of the *P. aeruginosa* cell envelope, yielding mean values of 15.9 ± 5.6 nm for OM-PG and 18.0 ± 5.1 nm for PG-IM (Fig. 4F and fig. S7), consistent with reported values (*49*). These values align with the membrane thicknesses extracted from each puncture event in the force curves (*W*_1_ = 17.0 ± 12.6 nm and *W*_2_ = 17.4 ± 12.0 nm) (Fig. 4, A and G).

**Fig. 5.**
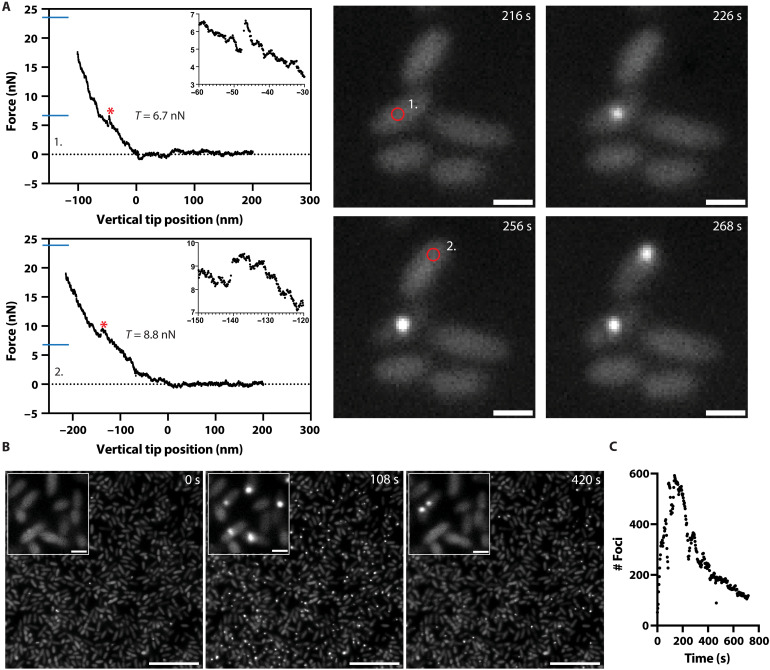
Both physical and chemical damage to the bacterial OM triggers H1-T6SS assembly. (**A**) The force threshold for membrane puncture (*) is shown for two indentation force curves. The median puncture forces *T*_1_ and *T*_2_ are indicated (blue lines; see Fig. 4B). Corresponding fluorescence images indicate the AFM tip position (red circle) and subsequent H1-T6SS assemblies. Scale bars, 1 μm. Applied force and loading rate are 15 nN and 10 μN/s, respectively, for both curves. (**B**) Representative time-lapse images capture the start, peak, and endpoint of H1-T6SS assembly, during incubation of *P. aeruginosa tssB1-mNeonGreen* in PMBN (40 μg/ml) in PBS where *t* = 0 s is the addition of PMBN. Scale bars, 10 μm. Inset: H1-T6SS assemblies resolved in individual cells. Scale bars, 1 μm. (**C**) Quantification of the H1-T6SS assembly rate is displayed as the number of TssB1-mNeonGreen foci over time.

To determine whether puncture of the first barrier (suggesting OM puncture) is sufficient to trigger H1-T6SS assembly, we analyzed ~100 force curves where the loading force just exceeded *T*_1_ (10 nN of force and 20 μN/s loading rate). Of these, 34 force curves showed a clear puncture (Fig. 5). Further analysis of the corresponding fluorescence microscopy images (Fig. 5A and fig. S8) showed that 88 % of such events resulted in H1-T6SS assembly. These data suggest that OM puncture is sufficient to trigger H1-T6SS assembly.

### Chemical disruption of the OM triggers H1-T6SS assembly

Previous studies show that external environmental factors can also trigger H1-T6SS assembly (*30*, *31*). For example, polymyxin B (PMB), which damages both the IM and OM of gram-negative bacteria (*50*), also induces H1-T6SS assembly (*31*). To further investigate whether OM damage alone could trigger H1-T6SS, we used a PMB-derivative PMB nonapeptide (PMBN), which disrupts the OM without affecting the IM (*51*). As above, *P. aeruginosa ∆retS*, *∆fliC*, *tssB1-mNeonGreen* cells were prepared on μ-dishes in the same manner as the AFM experiments and treated with PMBN (40 μg/ml) in phosphate-buffered saline (PBS) buffer. Within a minute of PMBN treatment, we observed increases in H1-T6SS assemblies (Fig. 5, B and C). The H1-T6SS activity was elevated for several minutes and slowly decreased over time (Fig. 5C). To compare this activity of PMBN to PMB, we performed additional control experiments on agarose pads as described in previous studies (*31*). Although both treatments led to an increase of H1-T6SS assembly, the response duration and intensity were lower for PMBN compared to that for PMB (fig. S9). We further note that the slower sample preparation required for the agarose pad experiments likely limited our observations to the decline phase of H1-T6SS assembly in both treatments. Last, we verified that the PMBN-triggered H1-T6SS assembly was dependent on the TagQRST-PpkA pathway by performing control experiments using the *P. aeruginosa ∆retS ∆fliC tssB1-mNeonGreen*, ∆*tagQ* and *P. aeruginosa ∆retS ∆fliC tssB1-mNeonGreen*, *∆ppkA* mutant strains. Neither strain showed an increase in H1-T6SS assembly upon PMBN treatment (40 μg/ml; fig. S10). This indicates that OM damage alone is sufficient to trigger H1-T6SS response and that this damage is sensed via the TagQRST-PpkA pathway.

## DISCUSSION

Our study underscores the sophisticated mechanisms that bacteria have evolved to dynamically assemble protein complexes in response to cell envelope damage. Through quantitative analysis, we show that indenting *P. aeruginosa* cells with forces greater than 35 nN punctures the cell envelope, resulting in an 80% H1-T6SS assembly response rate (Figs. 3 and 4). Lower forces between 5 and 15 nN can also initiate H1-T6SS assembly to a lesser degree (~40%) (Fig. 5A). Moreover, PMBN, which causes OM damage without killing target cells (*51*), can also trigger H1-T6SS assembly (Fig. 5B). Thus, *P. aeruginosa* has evolved a damage-dependent retaliatory response to attacks, with a response time of as little as 4 s to trigger H1-T6SS assembly (Fig. 2). Given that the average response time to H1-T6SS assembly is 13 s (Fig. 2) and that sheath assembly itself takes 9 s, we estimate a total time of ~20 s for *P. aeruginosa* to retaliate and deliver the first round of effectors into the attacking cell. This includes the time required to activate PpkA, phosphorylate Fha, initiate the assembly of the H1-T6SS membrane complex and baseplate, and polymerize and contract the sheath.

Quantifying the response of individual cells to single insults in mixed bacterial cultures has been challenging, given the difficulty in distinguishing which attacks by predator cells successfully target prey cells, such as between *P. aeruginosa* and T6SS^+^ *V. cholerae* (*27*, *52*). In this respect, correlative AFM-fluorescence imaging provides a powerful means to precisely time the response of individual *P. aeruginosa* cells to “attacks” from the AFM tip. A typical response involved multiple repeated H1-T6SS assemblies firing from the same site with a mean duration of 151 s but ranging widely from 10 to 507 s (Fig. 2). Hence, it may be less critical for cells to exert a tight control over the duration of their H1-T6SS response. Instead, maintaining a range of response times could be an evolved strategy to ensure sufficient toxin delivery during each attack.

Previous analysis demonstrated that killing is directional in mixtures of T6SS^+^ and T6SS^−^ prey bacteria, suggesting that H1-T6SS retaliation is precisely oriented toward attacking cells (*27*). This directional toxin delivery is determined by the precise spatial localization of the H1-T6SS membrane complex at the periphery of the rod-shaped cells. Here, our results show that the assembly of H1-T6SS is also targeted toward the incoming AFM tip (fig. S3). This indicates that the AFM tip closely mimics a bacterial attack thereby triggering a directed response. Studies have shown that the H1-T6SS sheath assembles perpendicular to the cell envelope (*16*, *53*). In this way, membrane damage incurred during an attack triggers localized H1-T6SS assembly at the damage site, followed by sheath contraction that pushes the Hcp tube and the VgrG/PAAR spike outward, directing toxin secretion toward the source of the attack. The combination of targeted H1-T6SS assembly and a rapid response to insults provides *P. aeruginosa* with a reloadable, precision weapon.

Unlike other bacteria, *P. aeruginosa* retaliates to incoming attacks with repeated assemblies of H1-T6SS (*45*). This is presumably achieved by delayed dephosphorylation by PppA after PpkA-mediated Fha phosphorylation, preventing further H1-T6SS assembly. Deleting PppA results in a continuous assembly of H1-T6SS and secretion of toxins; however, the overall efficiency of killing target cells is reduced (*27*). This could be due to the fact that stopping H1-T6SS assembly is needed to initiate new rounds of assemblies. We observed only limited numbers of assembly sites even during PMBN induced H1-T6SS assembly (Fig. 5 and movie S6). This suggests that cells need to maintain a low overall H1-T6SS assembly rate through PppA-mediated dephosphorylation of Fha to respond quickly and effectively to a new attack.

H1-T6SS is used by *P. aeruginosa* to retaliate against various attacks, such as other T6SSs or T4SSs (*27*, *31*, *54*). While specific T6SS effectors have been proposed to trigger H1-T6SS assembly during bacterial competition (*55*), H2-T6SS has been shown to activate H1-T6SS independently of effectors (*54*). However, it is also possible that other unknown signals could trigger H1-T6SS assembly. It was also suggested that lipases may activate H1-T6SS and that phospholipid hydrolysis could be the trigger for its assembly (*55*). Furthermore, EDTA treatments and interactions with DNA also show to trigger the H1-T6SS assembly (*30*). Therefore, it is conceivable that a common signal is generated by all these mechanisms. Through our study, we show that OM damage may be the common signal that activates the H1-T6SS during cell-cell interactions and chemical treatments.

Overall, our results link the damage induced response of H1-T6SS in *P. aeruginosa* to the TagQRST/PpkA pathway (Fig. 1, fig. S10, and movie S2). We propose a model whereby AFM tip–induced OM damage activates the IM-anchored PpkA by exposing it to an unknown signaling molecule normally confined to the OM. The activation of PpkA is likely due to its dimerization, as previous studies showed that artificial dimerization induces H1-T6SS assembly (*29*). The precise localization of the H1-T6SS machinery indicates that the signal diffuses over a limited area, thereby activating PpkA in close proximity to the damage site. The activated PpkA phosphorylates Fha, leading to repeated assemblies of the H1-T6SS and secretion of toxins through the T6SS baseplate and membrane complex. This localized assembly and rapid secretion of toxins results in effective retaliation against the attacking cell and provides *P. aeruginosa* an advantage in various ecological settings.

## MATERIALS AND METHODS

### Bacterial strains and growth conditions

Single colonies of *P. aeruginosa* PAO1 Δ*retS*, Δ*fliC*, *tssB1-mNeonGreen* were streaked on agar plates and grown overnight at 37°C. The overnight cultures were restreaked on fresh agar plates (Irgasan, 20 μg/ml) and grown for 2 hours at 37°C until an early logarithmic growth phase was reached. The cells were resuspended in LB to an optical density at 600 nm (OD_600nm_) of 0.25 and used for sample preparation immediately. The *P. aeruginosa* mutant strains were generated by allelic exchange using pEXG2 plasmid as described previously (*27*). The mNeonGreen tag was added to the C-terminus of *tssB1* with a linker encoding three alanine and three glycine residues. To delete *fliC*, the gene was fully deleted leaving only the first and last five residues. The ∆*tagQ* and ∆*ppkA* deletions were created in the same manner as the *∆fliC* mutant. The *V. cholerae* T6SS^+^ strain and its *∆tssM* mutant were described previously (*26*, *56*).

### Sample preparation

Resuspended cells (650 μl) were applied to ibiTreat-coated or glass-bottom 30-mm ø μ-dishes (ibidi) and incubated for 20 min. Then, the overstanding LB was slowly removed, and a new 650 μl of LB was applied to the μ-dish slowly to avoid washing off the attached cells. The wash step was repeated 3 times. Afterward, the μ-dishes were immediately used for imaging. During the experiments, fresh medium was applied about every 60 min to remove nonattached cells. For imaging of the PMBN (Sigma-Aldrich) effect, the same washing procedure was used. Right before imaging, instead of adding fresh LB to the sample, 650 μl of PMBN (40 μg/ml) in PBS was added to the μ-dish.

### Correlative AFM and fluorescence microscopy

The setup comprises of an epifluorescence/spinning disc confocal microscope (Visitron Systems GmbH, Germany) with an adapted sample stage upon which the AFM (NanoWizard I, JPK Instruments) is mounted. The entire system is placed on an active vibration isolation table (RS 2000, Newport). For fluorescence imaging, samples were excited by laser irradiation (488 nm, using a Sola Light Engine wide-field light source). The emission was collected by the same objective and imaged using a C11440 digital camera (Hamamatsu). A 100×, numerical aperture of 1.40 oil objective was used for time-lapse imaging.

A suitable region of interest was chosen by fluorescence imaging before force map acquisition, where arrays of 32 × 32 or 16 × 16 force distance curves were collected in areas spanning 30 μm by 30 μm or 15 μm by 15 μm. The area per pixel for a force curve is 0.9375 μm by 0.9375 μm, the *z*-length is 2 μm, and the recording time varies depending on the chosen loading rate and the spring constant of the chosen cantilever (0.5 to 2 s per pixel). The force-indentation curves were obtained at various loading rates of 4 to 20 μN/s and maximal forces of 5 to 100 nN. OLTESPA R3 (Bruker) cantilevers with a nominal spring constant of 2 N/m were used in all force-mapping experiments. Cantilever spring constants were determined using the thermal noise method (in air) before each experiment. Corresponding time-lapse fluorescence images were collected at 250-ms exposure time at time intervals of 1 to 3 s. For each correlative AFM-fluorescence microscopy experiment, the fluorescence time-lapse was started before the initiation of the AFM force map to ensure that any assemblies triggered by AFM tip indentation were imaged before the actual AFM trigger. As a control, we also examined fluorescence movies for the baseline H1-T6SS activity of our strain by quantifying the number of assemblies in a 30 μm–by–30 μm area prior to AFM indentation over a time of 80 s (Fig. 3A). For the PMBN assay, the cells where imaged immediately after PMBN was added using the same microscope and imaging conditions.

### Cryo–electron microscopy

Cells were fixed using 5% glutaraldehyde before imaging. A 4-μl aliquot of bacteria suspension was adsorbed onto a glow-discharged (Quorum, UK) holey carbon-coated grid (Lacey, Ted Pella, USA), blotted 4 s with Whatman 1 filter paper and vitrified into liquid ethane at −180°C using a Leica GP2 plunger (Leica microsystems, Austria). The frozen grids were transferred onto a Titan Krios Electron microscope (FEI, USA) operating at 300 kV and equipped with a GIF Quantum LS Imaging Filter. Micrographs were collected on K2 direct detector from Gatan using low-dose system (20 electrons/Å^2^) at a magnification of ×42,000 corresponding to 3.4 Å per pixel on the image. Defocus values were −4 to −5 μm.

### Data analysis

Fluorescence data were acquired with VisiView (VisiLab) and processed in ImageJ. The kymographs were built with the Kymograph Builder plugin. To that end, line segments of bacterial cells showing fluorescent foci were measured, and, using the plugin, the line segment of each frame of the time-lapse series was merged into one continuous kymograph. A gray-scale plot was performed on each of these kymographs to obtain the corresponding fluorescence intensity plot profiles. H1-T6SS assemblies and cells were segmented and counted using TrackMate. The number of cells was measured using the thresholding detector of TrackMate, and the fluorescent foci were detected using the LoG detector with a Quality factor of 5000 and a spot size of 0.4-μm diameter. Response times were counted by looking at the number of frames between AFM indentation and increase in fluorescence intensity at the same location. AFM force data were preprocessed with the JPK Data Processing software and converted into .csv files for analysis using a custom Python script. Preprocessing for all curves included: adjusting baseline and contact point to zero and converting cantilever deflection versus *z*-piezo distance into force-indentation curves. Further analysis and Young’s modulus calculation were done with Origin (OriginLab 2016) using the modified Hertz-Sneddon contact model (*42*). Data representation and graphs were done in Prism.

### Pressure calculation

The pressure exerted by the AFM cantilever tip was calculated using the following formula (*42*)

$$p=\frac{F}{{\text{A}}_{\text{c}}\left(h\right)}=\frac{F}{h^{2}\text{tan}\;\text{FA}\;\left(\text{tan}\;\text{BA}\;+\text{tan}\;\text{SA}\right)}$$ (1)

where *p* is the pressure, *F* is the loading force, and A_c_(*h*) is the area of contact as a function of the indentation depth *h*. The area of contact for a three-sided pyramidal indenter can be further split into the indentation depth squared and tangents of the front angle (FA), back angle (BA), and side angle (SA) of the pyramid.

### Fluorescence live-cell imaging of *P. aeruginosa* on agarose pads

*P. aeruginosa* PAO1 Δ*retS tssB1-mNeonGreen* lawns were grown overnight at 37°C on LB agar plates without antibiotics. The following morning, cells were gathered and spread on a fresh LB agar plate and incubated at 37°C for 2 hours. Cells were gathered again, washed once with LB agar, and diluted to a concentration of OD_600nm_ of 10. The cultures (1.2 μl) were spotted on LB pads consisting of two-thirds of PBS and one-third of LB with 1% agarose. For imaging of PMB and PMBN effect on H1-T6SS activation, peptides were added directly to the pad before solidification. The microscope setup was used as previously described (*57*, *58*) with images collected every 3 s for 2 min for an exposure time of 150 ms on the green fluorescent protein channel.
