# Supplementary material for: Pseudomonas aeruginosa assembles H1-T6SS in response to physical and chemical damage of the outer membrane
Source: Sci Adv. 2025 Mar 5;11(10):eadr1713. doi: 10.1126/sciadv.adr1713 (PMC11881912; doi:10.1126/sciadv.adr1713)
Supplement: Supplementary file 1 — Figs. S1 to S10 Legends for movies S1 to S6 [file sciadv.adr1713_sm.pdf]

Science Advances
AAAS

Supplementary Materials for

Pseudomonas aeruginosa assembles H1-T6SS in response to physical and chemical damage of the outer membrane

Mitchell Brüderlin *et al.*

Corresponding author: Roderick Y. H. Lim, roderick.lim@unibas.ch; Marek Basler, marek.basler@unibas.ch

Sci. Adv. **11**, eadr1713 (2025)
DOI: 10.1126/sciadv.adr1713

The PDF file includes:

Figs. S1 to S10
Legends for movies S1 to S6

Other Supplementary Material for this manuscript includes the following:

Movies S1 to S6

Supplementary Materials

Supplementary Figures

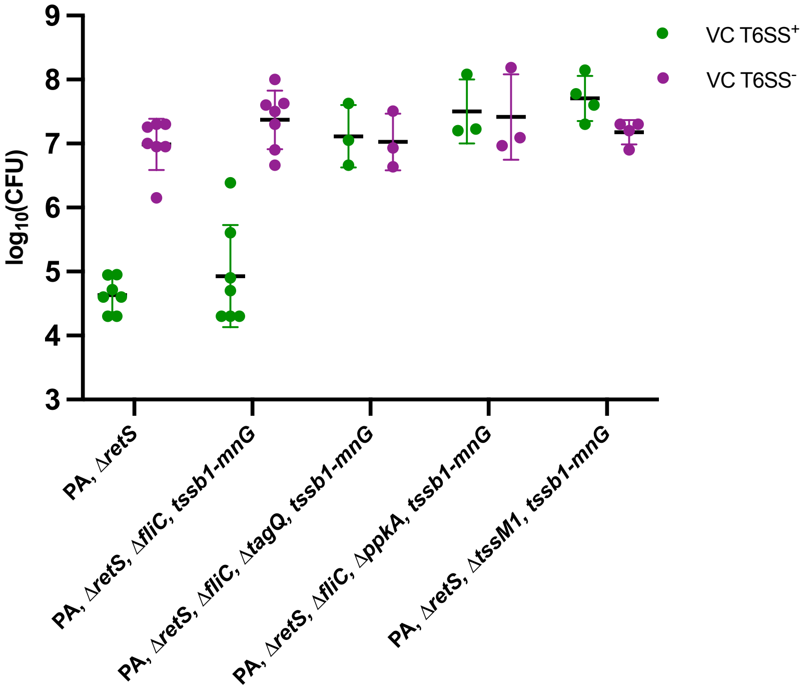

Supplementary Figure 1. Competition of *P. aeruginosa* with *V. cholerae*. The plot depicts the *V. cholerae* (VC) recovery after competition with the three *P. aeruginosa* (PA) strains used in this study as well as *P. aeruginosa* (PA), Δ*retS* as positive control and *P. aeruginosa* (PA), Δ*retS*, Δ*tssM1, tssb1-mng* as a negative control. The recovery upon competition with T6SS^{+} *V. cholerae* is shown in purple and T6SS^{-} *V. cholerae* in green.

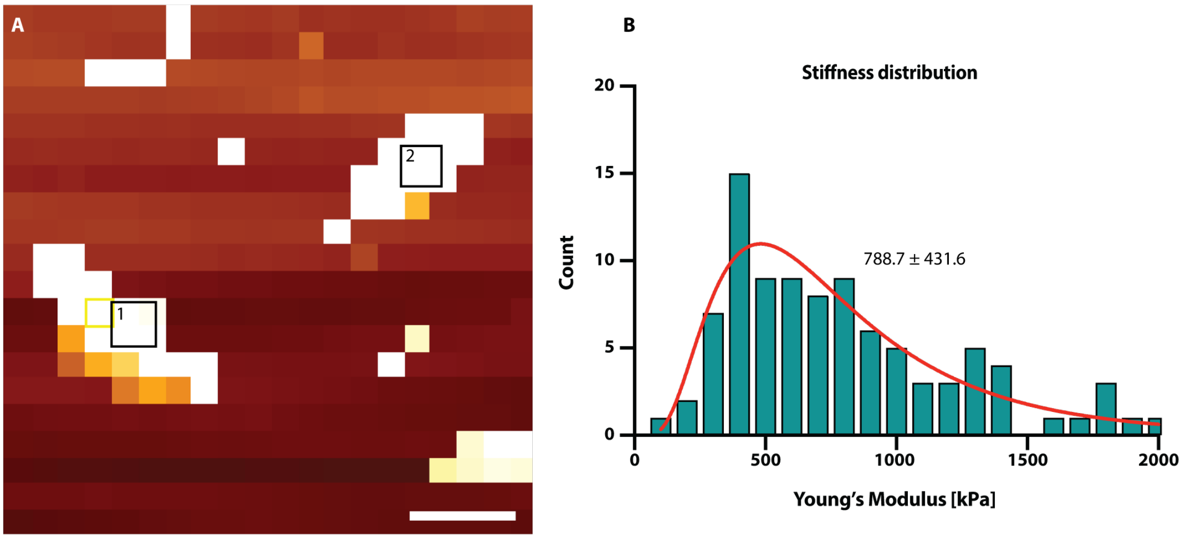

Supplementary Figure 2. Single cell force mapping. (A) Overview force-map comprising a 20 x 20 force curves (pixels) acquired using and indentation force of 0.5 nN within a 5 x 5 μm^2 area. Black boxes indicate 2 regions where 64 force curves were collected over 300 nm x 300 nm area with an 8 x 8 grid. Scale bar indicates 1 μm. **(B)** All force curves of individual cells (N = 2, n = 115) were recorded and the Youngs modulus distribution displayed as a bar plot. The Young's modulus was fitted using the Hertz-Sneddon method and the distribution was analyzed using a log normal distribution fit resulting in the displayed mean contact stiffness.

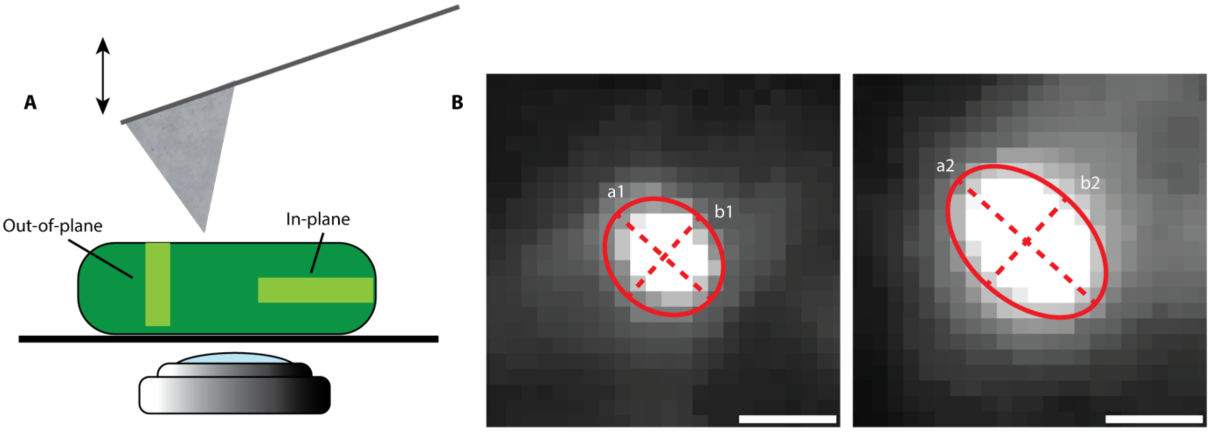

Supplementary Figure 3: Analysis of orientation of H1-T6SS assemblies. (A) Scheme indicating two possible assembly directions of the T6SS (light green) within *P. aeruginosa*. On the left an out of plane assembly points towards the AFM tip and to the right an in-plane H1-T6SS assembly. **(B)** The red ellipses show the outline of two fluorescent foci (TssB1-mNeonGreen). The major axis (a1 and a2) and minor axis (b1 and b2) are shown as red dotted lines and labelled accordingly. The ratios (a/b) for these two examples are 1.44 (in plane assembly) and 1.13 (out of plane assembly) respectively. Scale bar, 400 nm.

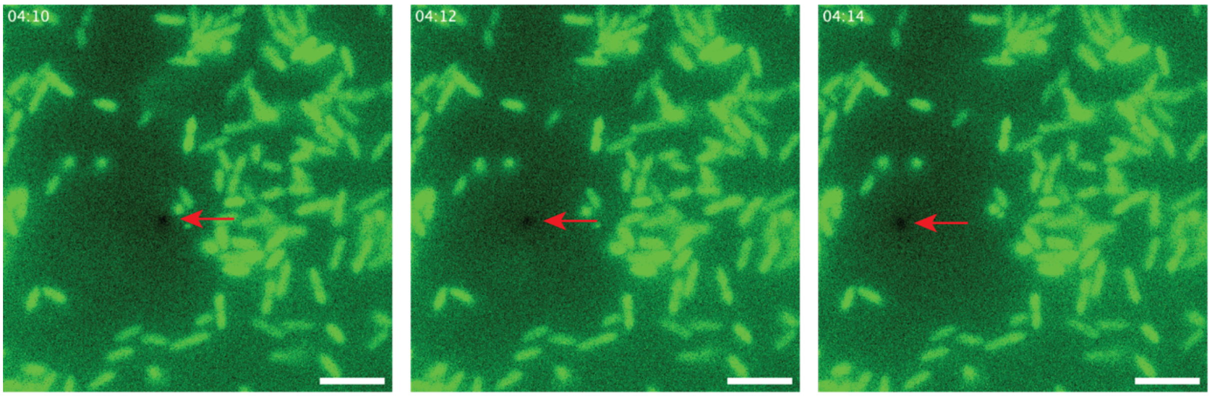

Supplementary Figure 4: Capturing AFM tip position in fluorescence images. The position of the AFM can be determined by localizing small regions of decreased fluorescence as well as the movement of cells caused by the passing AFM tip (see Supplementary Movie 3). The red arrows show three consecutive frames where the position of the AFM can be directly determined. Scale bar, 5 µm.

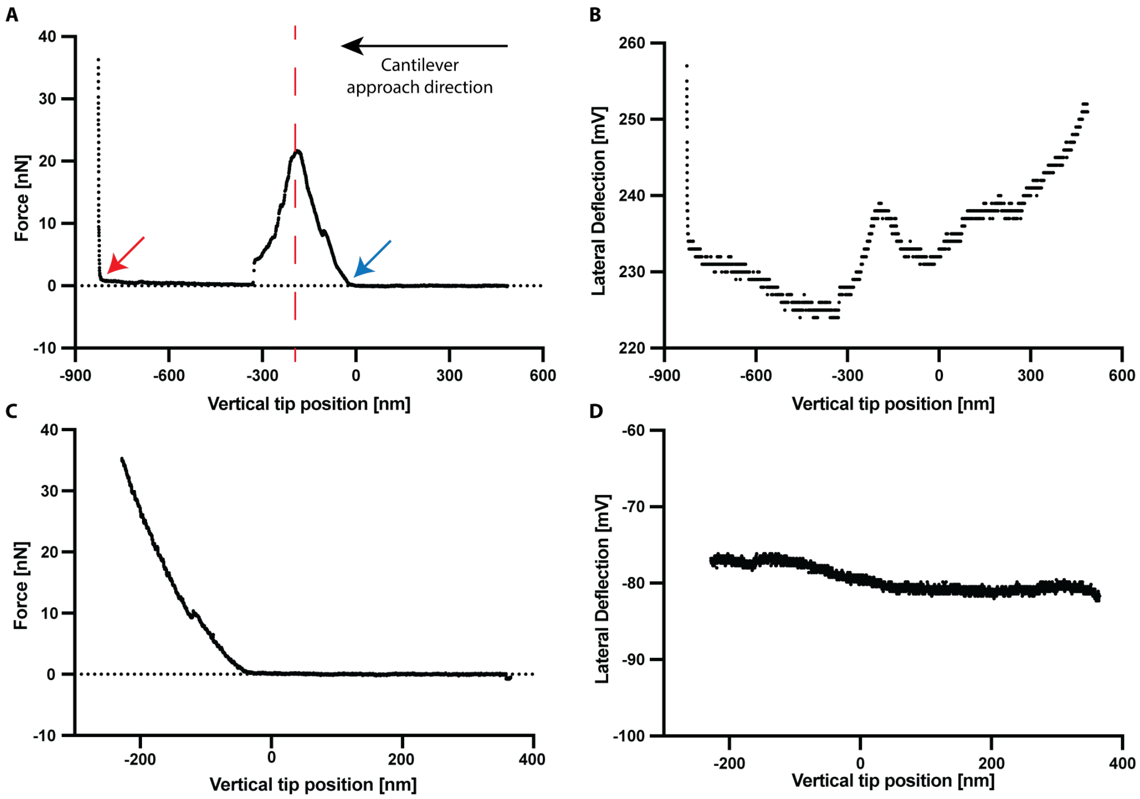

Supplementary Figure 5: Occasional displacement of cells by force measurements. (A) The force curve was recorded at a sampling rate of 10’000 Hz with a loading rate of 20 µN/s and a maximum force of 35 nN. The blue arrow indicates the contact point with the bacterial cell leading to the start of an indentation curve. The red dashed line indicates the point where the cell was pushed aside, resulting in a drop of measured force. The red arrow indicates the contact point with the stiff glass bottom surface of the µ-dish. **(B)** The lateral deflection mimics the deflection in vertical as the cell is hit at an angle. **(C)** A successful indentation is shown for comparison. The curve was recorded at a sampling rate of 10’000 Hz with a loading rate of 20 µN/s and a maximum applied force of 35 nN. **(D)** Lateral deflection of the indentation in C.

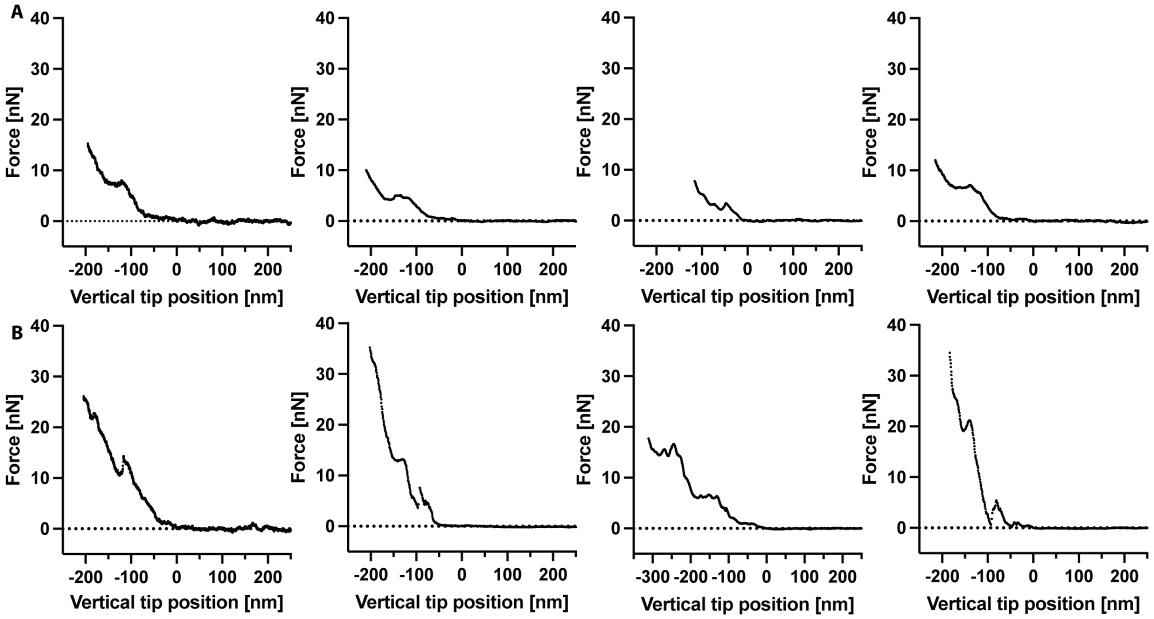

Supplementary Figure 6: Force curves depicting single and double puncturing events. Each force curve is shown as a plot of cantilever deflection vs. vertical tip position. **(A)** Force curves showing one clear puncture event before reaching the maximum applied force. The applied force and loading rate are as follows, from left to right: (15 nN, 20 μN/s), (10 nN, 20 μN/s), (5 nN, 10 μN/s) and (15 nN, 20 μN/s). **(B)** Force curves showing two distinct puncture events. The applied force and loading rate are as follows, from left to right: (25 nN, 20 μN/s), (35 nN, 10 μN/s), (15 nN, 20 μN/s) and (35 nN, 20 μN/s).

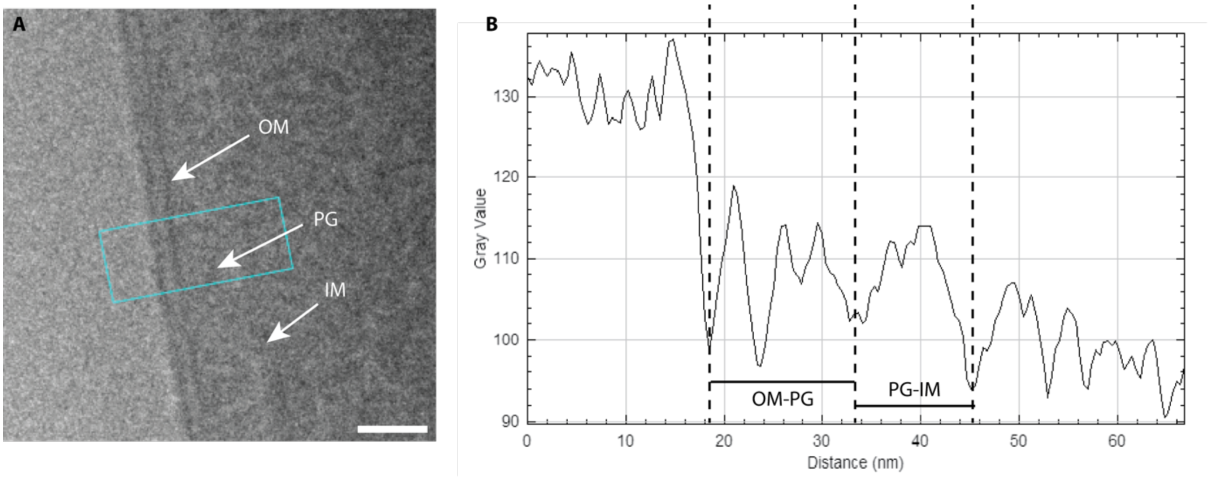

Supplementary Figure 7: Cryo-EM visualization of the *P. aeruginosa* cell envelope. (A) Cryo-EM image showing a cross-section of the *P. aeruginosa* ∆*retS,* ∆*fliC, tssB1-mNeonGreen* cell envelope. The rectangular ROI used for the thickness analysis is highlighted in cyan. Scale bar, 20 nm. **(B)** Average line profile with gray values on the y-axis and distance on the x-axis. Distinct layers in the cell envelope layers are visible due to their higher density, which results in decrease in grey values on the plot profile. The OM-PG and PG-IM distances are indicated by the dotted black lines and labelled.

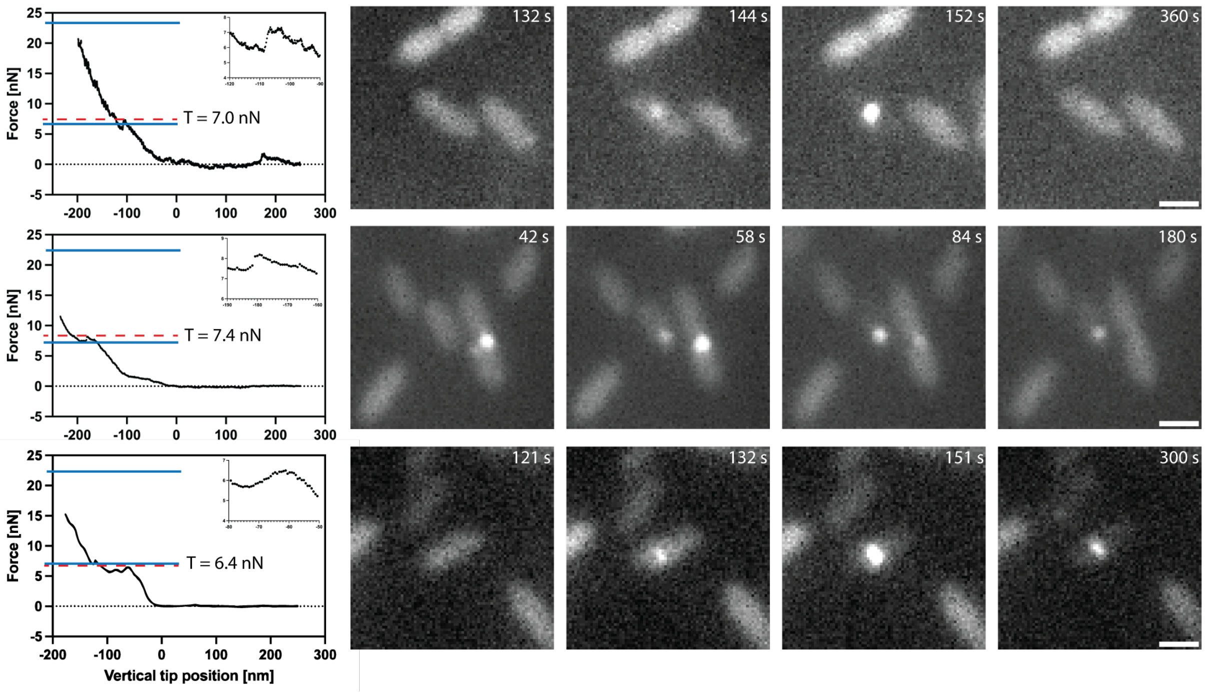

Supplementary Figure 8: Outer membrane damage triggers H1-T6SS assembly. Each row shows the force curve of a successful AFM indentation and the corresponding T6SS assembly. The puncture force for each curve is displayed as the red dashed line and the force needed for the break is indicated. The median puncture forces T_1 = 6.8 nN and T_2 = 23.9 nN are indicated by the blue lines. The applied forces and loading rates are, from top to bottom: (20 nN, 4 uN/s), (10 nN, 20 µN/s) and (15 nN, 20 µN/s). The fluorescence images show frames corresponding to, from left to right: 1. AFM indentation, 2. detection of a TssB1-mNeonGreen focus, 3. peak of fluorescence signal for each focus, 4. end of the time lapse. Scale bar, 1 µm.

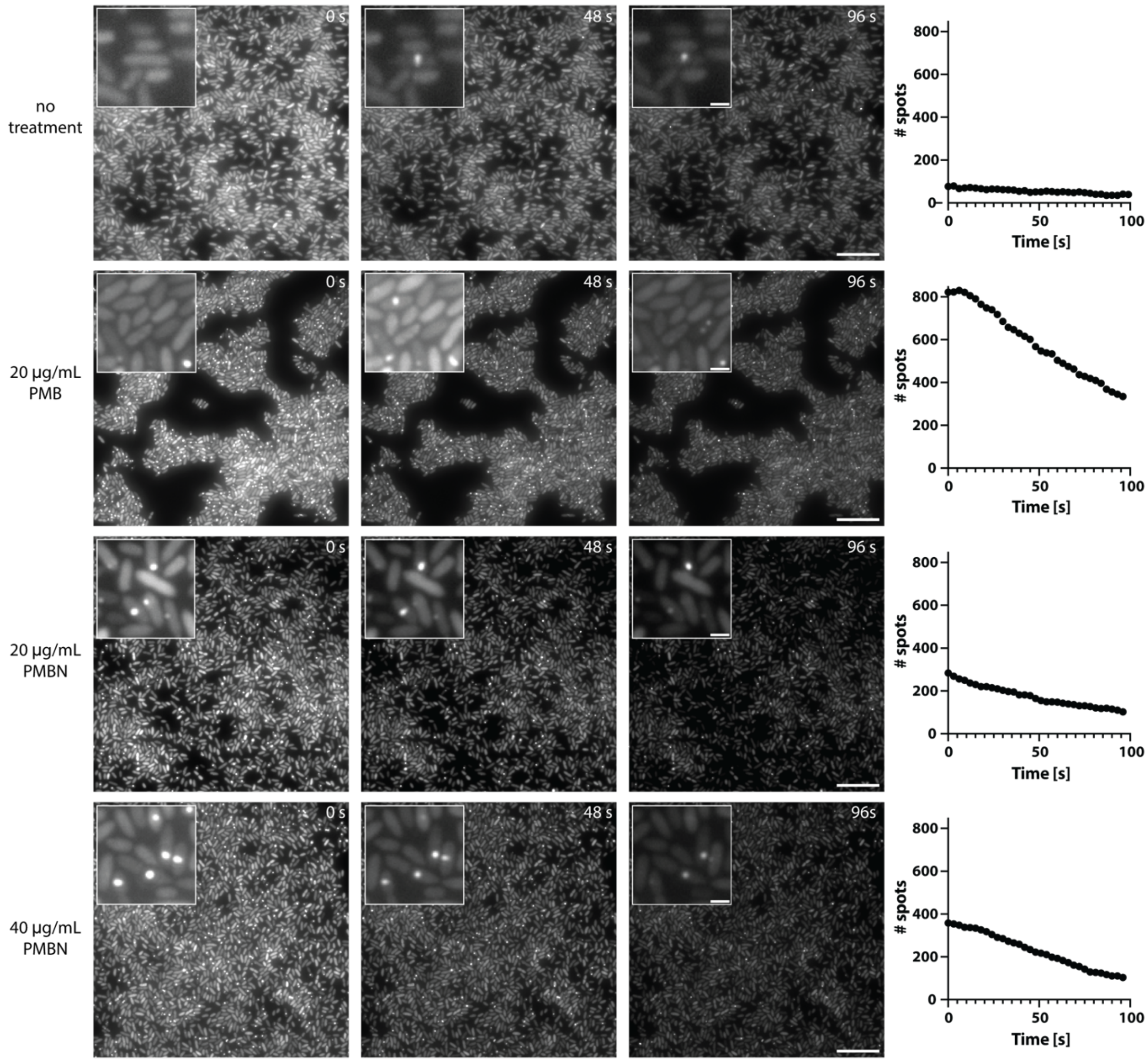

Supplementary Figure 9: Triggering of the H1-T6SS assembly by chemically induced membrane damage. Fluorescence imaging of *P. aeruginosa tssB1-mNeonGreen* cells on agarose pads. Each condition was imaged over 100 seconds. Each row represents one condition (untreated, or agarose pads containing 20 µg/mL PMB, 20 µg/mL PMBN and 40 µg/mL PMBN). For each condition 3 representative images show the change in the number of the fluorescent foci over time (TssB1-mNeonGreen). Scale bars, 10 µm and 1 µm for the overview image and the inset respectively. The graphs display the number of foci for each frame of the analyzed time-lapse.

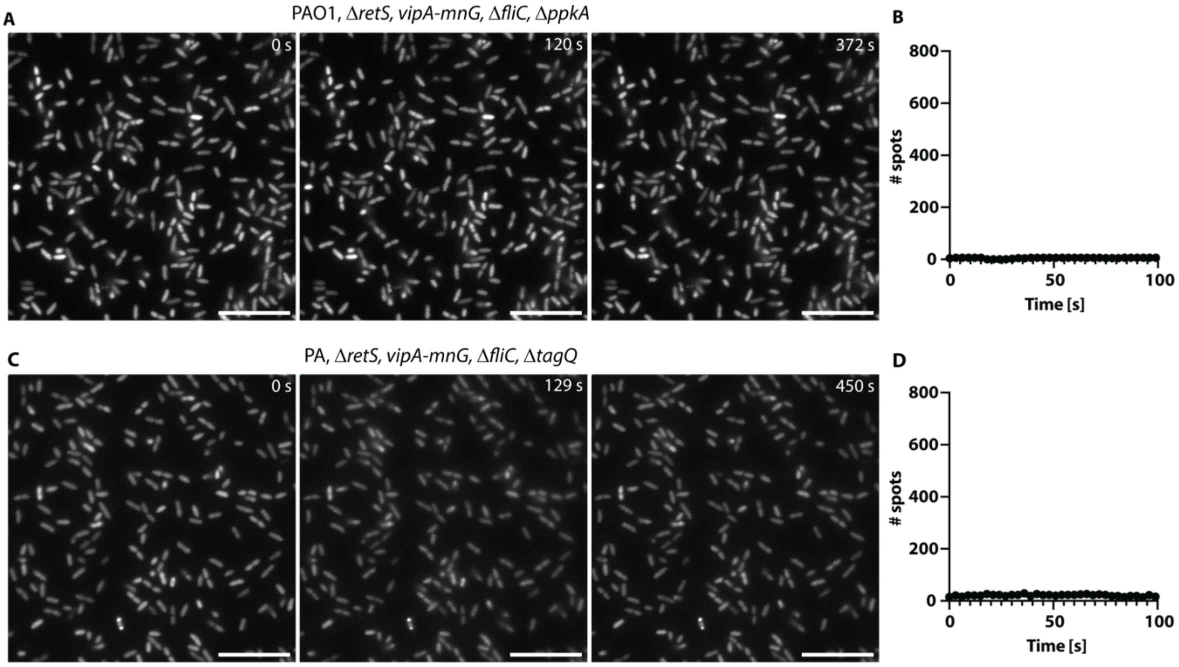

Supplementary Figure 10. PpkA and TagQ are required for PMBN induced membrane damage. **(A)** Representative timelapse images capture start, middle and endpoint of H1-T6SS assembly, during incubation of *P. aeruginosa ΔretS, ΔfliC, tssB1-mNeonGreen*, *ΔppkA* with 40 µg/ml PMBN in PBS. Scale bar, 10 µm. **(B)** Quantification of the H1-T6SS assembly rate, detected as TssB1-mNeonGreen foci over time. **(C)** Representative timelapse images capture the start, middle and endpoint of H1-T6SS assembly, during incubation of *P. aeruginosa ΔretS, ΔfliC, tssB1-mNeonGreen, ΔtagQ* with 40 µg/ml PMBN in PBS. Scale bar, 10 µm. **(D)** Quantification of the H1-T6SS assembly rate, detected as TssB1-mNeonGreen foci over time.

Supplementary Movies Legends

Movie S1. Overlay of *P. aeruginosa tssB1-mNeonGreen ΔretS ΔfliC* with the corresponding force map. This video shows a fluorescence time lapse at the top and the corresponding AFM force map below. H1-T6SS assemblies are visualized with a TssB1-mNeonGreen fusion protein and are shown in green. The blue dot on the AFM force map indicates the position of the AFM tip over time. Scale bar, 5 μm

Movie S2. Force mapping experiment with a *P. aeruginosa tssB1-mNeonGreen ΔretS ΔfliC ΔppkA* sample shows expression of TssB1-mNeonGreen without any H1-T6SS activity. Cell movement indicates successful indentation. TssB1-mNeonGreen fluorescence is shown in gray scale. Scale bar, 10 μm

Movie S3. Increasing the contrast in a *P. aeruginosa tssB1-mNeonGreen ΔretS ΔfliC* fluorescence time-lapse during a force-mapping experiment shows the position of the AFM tip as a dark spot (relating to Fig. S4) and the cantilever as a dark moving shadow. TssB1-mNeonGreen fluorescence is shown in green. The time-lapse was recorded over the course of 9.5 minutes every 2 seconds. Scale bar, 5 μm

Movie S4. Consecutive time-lapse movies show H1-T6SS activity of *P. aeruginosa tssB1-mNeonGreen ΔretS ΔfliC* in response to the following forces: 100, 70, 35, 15, 10 and 5 nN at 20 μN/s. The runtime of each experiment is shown in the upper left corner. The loading force is indicated in the upper right corner. Scale bar, 5 μm

Movie S5. Force mapping experiment with increased cell density *P. aeruginosa tssB1-mNeonGreen ΔretS ΔfliC* sample shows increased H1-T6SS response rate at lower forces. The timelapse was recorded over 3.33 minutes at a rate of 1 frame per second. The force applied by the AFM tip was 5 nN at a loading rate of 20 μN/s using a grid size of 16 x 16 on an area of 15 x 15 μm^2.

Movie S6. The movie displays H1-T6SS activity in response to 40 μg/ml PMBN treatment in *tssB1-mNeonGreen ΔretS ΔfliC.* H1-T6SS assemblies are visualized with a TssB1-mNeonGreen fusion protein and are shown in gray scale. The scale bar indicates 10 μm. PMBN is added 20 s before imaging starts. H1-T6SS response is monitored over 732 s with 3 s/frame.
